# Supplementary material for: Multi‐dataset identification of innovative feature genes and molecular mechanisms in keratoconus
Source: J Cell Mol Med. 2024 Sep 19;28(18):e70079. doi: 10.1111/jcmm.70079 (PMC11412914; doi:10.1111/jcmm.70079)
Supplement: Supplementary file 2 — Material S2. [file JCMM-28-e70079-s002.zip › 7.GSEA analysis/1.ARL11/KEGG_SNARE_INTERACTIONS_IN_VESICULAR_TRANSPORT.html]

Details for gene set KEGG\_SNARE\_INTERACTIONS\_IN\_VESICULAR\_TRANSPORT[GSEA]

|  || Dataset | ARL11.group.cls#h\_versus\_l |
| Phenotype | group.cls#h\_versus\_l |
| Upregulated in class | l |
| GeneSet | KEGG\_SNARE\_INTERACTIONS\_IN\_VESICULAR\_TRANSPORT |
| Enrichment Score (ES) | -0.33402124 |
| Normalized Enrichment Score (NES) | -1.1486273 |
| Nominal p-value | 0.30170777 |
| FDR q-value | 1.0 |
| FWER p-Value | 0.991 |
Table: GSEA Results Summary

  

Fig 1: Enrichment plot: KEGG\_SNARE\_INTERACTIONS\_IN\_VESICULAR\_TRANSPORT      
 Profile of the Running ES Score & Positions of GeneSet Members on the Rank Ordered List

  

| SYMBOL | TITLE | RANK IN GENE LIST | RANK METRIC SCORE | RUNNING ES | CORE ENRICHMENT || 1 | STX2 | na | 7423 | 0.103 | -0.1580 | No |
| 2 | VAMP1 | na | 10601 | 0.052 | -0.2212 | No |
| 3 | STX19 | na | 10624 | 0.052 | -0.1936 | No |
| 4 | GOSR1 | na | 11451 | 0.042 | -0.1946 | No |
| 5 | GOSR2 | na | 11653 | 0.040 | -0.1789 | No |
| 6 | SNAP25 | na | 12779 | 0.029 | -0.1957 | No |
| 7 | VAMP2 | na | 13168 | 0.026 | -0.1930 | No |
| 8 | VAMP8 | na | 13737 | 0.021 | -0.1981 | No |
| 9 | YKT6 | na | 14682 | 0.014 | -0.2179 | No |
| 10 | STX12 | na | 15222 | 0.010 | -0.2283 | No |
| 11 | VAMP5 | na | 16089 | 0.003 | -0.2516 | No |
| 12 | STX6 | na | 16103 | 0.003 | -0.2503 | No |
| 13 | VAMP3 | na | 16186 | 0.002 | -0.2514 | No |
| 14 | BET1 | na | 16329 | 0.001 | -0.2547 | No |
| 15 | STX16 | na | 16711 | -0.001 | -0.2649 | No |
| 16 | SEC22B | na | 18136 | -0.011 | -0.2998 | No |
| 17 | TSNARE1 | na | 18842 | -0.016 | -0.3115 | No |
| 18 | BET1L | na | 18899 | -0.016 | -0.3043 | No |
| 19 | VTI1B | na | 19254 | -0.019 | -0.3044 | No |
| 20 | STX8 | na | 20172 | -0.025 | -0.3174 | No |
| 21 | STX4 | na | 20750 | -0.029 | -0.3181 | Yes |
| 22 | STX7 | na | 21239 | -0.033 | -0.3143 | Yes |
| 23 | STX17 | na | 21247 | -0.033 | -0.2965 | Yes |
| 24 | STX5 | na | 21276 | -0.033 | -0.2793 | Yes |
| 25 | VTI1A | na | 21694 | -0.037 | -0.2714 | Yes |
| 26 | VAMP7 | na | 22115 | -0.041 | -0.2613 | Yes |
| 27 | USE1 | na | 22361 | -0.044 | -0.2448 | Yes |
| 28 | STX10 | na | 22766 | -0.048 | -0.2307 | Yes |
| 29 | STX18 | na | 23908 | -0.061 | -0.2306 | Yes |
| 30 | STX1B | na | 24087 | -0.064 | -0.2013 | Yes |
| 31 | SNAP47 | na | 24476 | -0.069 | -0.1751 | Yes |
| 32 | BNIP1 | na | 25096 | -0.077 | -0.1512 | Yes |
| 33 | VAMP4 | na | 25113 | -0.077 | -0.1097 | Yes |
| 34 | SNAP29 | na | 26887 | -0.106 | -0.1034 | Yes |
| 35 | STX11 | na | 28189 | -0.136 | -0.0671 | Yes |
| 36 | SNAP23 | na | 28314 | -0.139 | 0.0044 | Yes |
| 37 | STX3 | na | 28829 | -0.153 | 0.0723 | Yes |
| 38 | STX1A | na | 29766 | -0.180 | 0.1430 | Yes |
Table: GSEA details [plain text format]

  

Fig 2: KEGG\_SNARE\_INTERACTIONS\_IN\_VESICULAR\_TRANSPORT      
 Blue-Pink O' Gram in the Space of the Analyzed GeneSet

  

Fig 3: KEGG\_SNARE\_INTERACTIONS\_IN\_VESICULAR\_TRANSPORT: Random ES distribution      
 Gene set null distribution of ES for **KEGG\_SNARE\_INTERACTIONS\_IN\_VESICULAR\_TRANSPORT**
